# The appropriate sample-handling procedure for measuring the plasma β-amyloid level using a fully automated immunoassay

**DOI:** 10.1038/s41598-024-65264-1

**Published:** 2024-06-20

**Authors:** Kengo Ishiki, Kazuto Yamashita, Shunsuke Watanabe, Masahiro Miura, Junko Kawahira, Yuji Arimatsu, Kana Kawasaki, Shigeki Iwanaga, Toshiyuki Sato

**Affiliations:** 1 Central Research Laboratories, Sysmex Corporation, 4-4-4 Takatsukadai, Nishi-Ku, Kobe, 651-2271 Japan; 2 Reagent Engineering, Sysmex Corporation, 4-4-4 Takatsukadai, Nishi-Ku, Kobe, 651-2271 Japan

**Keywords:** Diagnostic markers, Dementia

## Abstract

Plasma β-amyloid (Aβ) assays are a promising tool for Alzheimer’s disease diagnosis in clinical practice. To obtain reliable results, establishing an appropriate sample-handling procedure for each analytical platform is warranted. This study proposes an appropriate sample-handling procedure using HISCL analyzer by elucidating the individual/combined effects of pre-analytical parameters on plasma Aβ42/Aβ40 levels. We investigated the effects of various pre-analytical parameters, including storage times for whole blood, plasma, and freezing conditions, on plasma Aβ42/Aβ40 levels, and confirmed if these values met the acceptable criteria. Plasma Aβ42/Aβ40 levels were acceptable in all conditions. We determined our protocol by confirming that plasma Aβ42/Aβ40 levels remained acceptable when combining pre-analytical parameters. We established an appropriate sample-handling protocol that ensures reliable measurement of plasma Aβ42/Aβ40 levels using HISCL analyzer. We believe the Aβ assay, with our protocol, shows promise for aiding AD diagnosis in clinical settings.

## Introduction

Alzheimer’s disease (AD) is the most common form of dementia. The condition causes memory and cognitive decline and/or interference with activities of daily living. The number of patients with dementia is estimated to increase to over 150 million by 2050^1^, and AD is involved in 60–70% of dementia cases^2^. Treatments for AD have been limited to symptomatic drugs with modest benefits^3^. Therefore, there is an urgent need to develop drugs that have the potential to change the progression of AD.

A main pathological feature of AD is the accumulation of β-amyloid (Aβ) as amyloid plaques in the brain^4,5^. The accumulation of Aβ induces toxic damage, resulting in synaptic dysfunction and subsequent neurodegeneration^6,7^. Many clinical trials have been conducted to develop anti-Aβ immunotherapies that remove these amyloid plaques^8–11^. In 2021, the US Food and Drug Administration granted accelerated approval of aducanumab as the first disease-modifying therapy for patients with mild AD and mild cognitive impairment due to AD^12^. Recently, another anti-Aβ monoclonal antibody, lecanemab, showed critical evidence of slowing cognitive decline and was also granted accelerated approval^13^. The appropriate use recommendations for aducanumab published by an expert panel suggest that Aβ positivity be confirmed prior to treatment initiation^14^. The prescribing information for lecanemab also requires confirmation of brain Aβ pathology for treatment. Therefore, along with the widespread administration of these promising drugs to patients, there is an urgent need to establish a method to detect brain Aβ pathology in routine clinical practice.

Amyloid positron emission tomography (PET) and/or cerebrospinal fluid (CSF) testing are conventional techniques used to detect amyloid pathology. However, their capacity is limited in routine clinical practice due to their accessibility, cost, and required human resources^15,16^. Blood-based biomarkers, combined with PET and CSF tests, are expected to be promising tools for assisting in AD diagnosis in clinical practice^17–20^. Particularly, the level of plasma Aβ_1–42_ (Aβ42) to Aβ_1–40_ (Aβ40) is associated with brain Aβ pathology, and mounting evidence has shown that this level is highly concordant with the amyloid PET status obtained using various analytical platforms^21–26^. We previously reported the development of plasma Aβ assays that showed sufficiently high performance for predicting brain Aβ pathology defined by amyloid PET^26^. To obtain consistent results and accelerate the implementation of these promising plasma Aβ assays in clinical practice, standardization of the measurement protocol is required^19,20^.

Recent studies have focused on the effects of pre-analytical parameters on biomarker measurement, including changes in the plasma Aβ42/Aβ40 level^27–31^. Plasma Aβ peptides are unstable molecules; therefore, their measurement is affected by pre-analytical parameters, for instance, external factors such as sample handling (e.g., whole blood and plasma storage conditions) and analytical platforms. There are various Aβ assays across different analytical platforms that target different epitopes of Aβ peptides, and the procedure for handling samples may vary, including differences in stirring speed and reaction time/temperature^27,29^. To obtain reliable results, a standard sample-handling procedure should be provided for each platform. Furthermore, many studies have only investigated the effects of individual pre-analytical factors by varying one parameter during sample handling and assessing this against each reference condition; consequently, interactions between parameters that may affect plasma Aβ42/Aβ40 levels have not been considered. The effects of certain parameters are known to combine additively or synergistically^32^. Whether combined effects of these parameters on the plasma Aβ42/Aβ40 level occur is unknown. Therefore, protocols proposed by previous studies should be determined by evaluating the combined effects of pre-analytical parameters.

**Figure 1:**
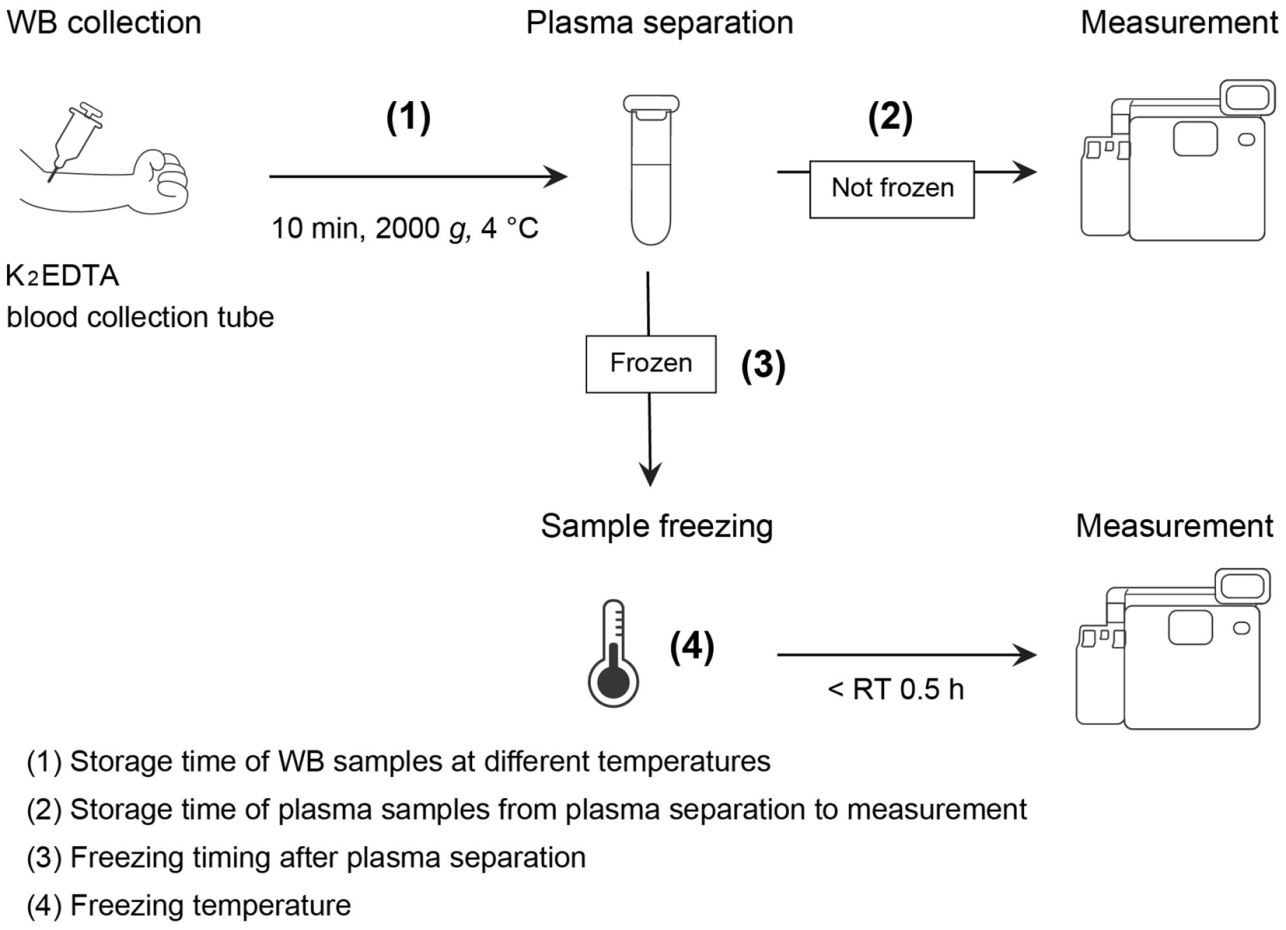
Schematic illustration of a sample-handling procedure from WB collection to measurement of plasma Aβ42/Aβ40 levels. The following parameters were assessed: (1) storage time of WB samples at different temperatures, (2) storage time of plasma samples from plasma separation to measurement, (3) freezing timing after plasma separation, and (4) freezing temperature. EDTA, ethylenediaminetetraacetic acid; WB, whole blood; RT, room temperature; Aβ, β-amyloid.

In this study, we first elucidated the effects of pre-analytical parameters, including sample storage time/temperature and freezing conditions (Fig. 1), on plasma Aβ42/Aβ40 levels measured by the HISCL™-5000/HISCL™-800 (HISCL analyzer). The HISCL analyzer is a fully automated chemiluminescence enzyme immunoassay that is widely used in clinical practice due to its rapid reaction time and high reproducibility. Finally, we established an appropriate sample-handling procedure using the HISCL analyzer by evaluating the combined effects of individual pre-analytical parameters on the plasma Aβ42/Aβ40 level.

## Results

### Effects of WB and plasma storage time and temperature

**Figure 2:**
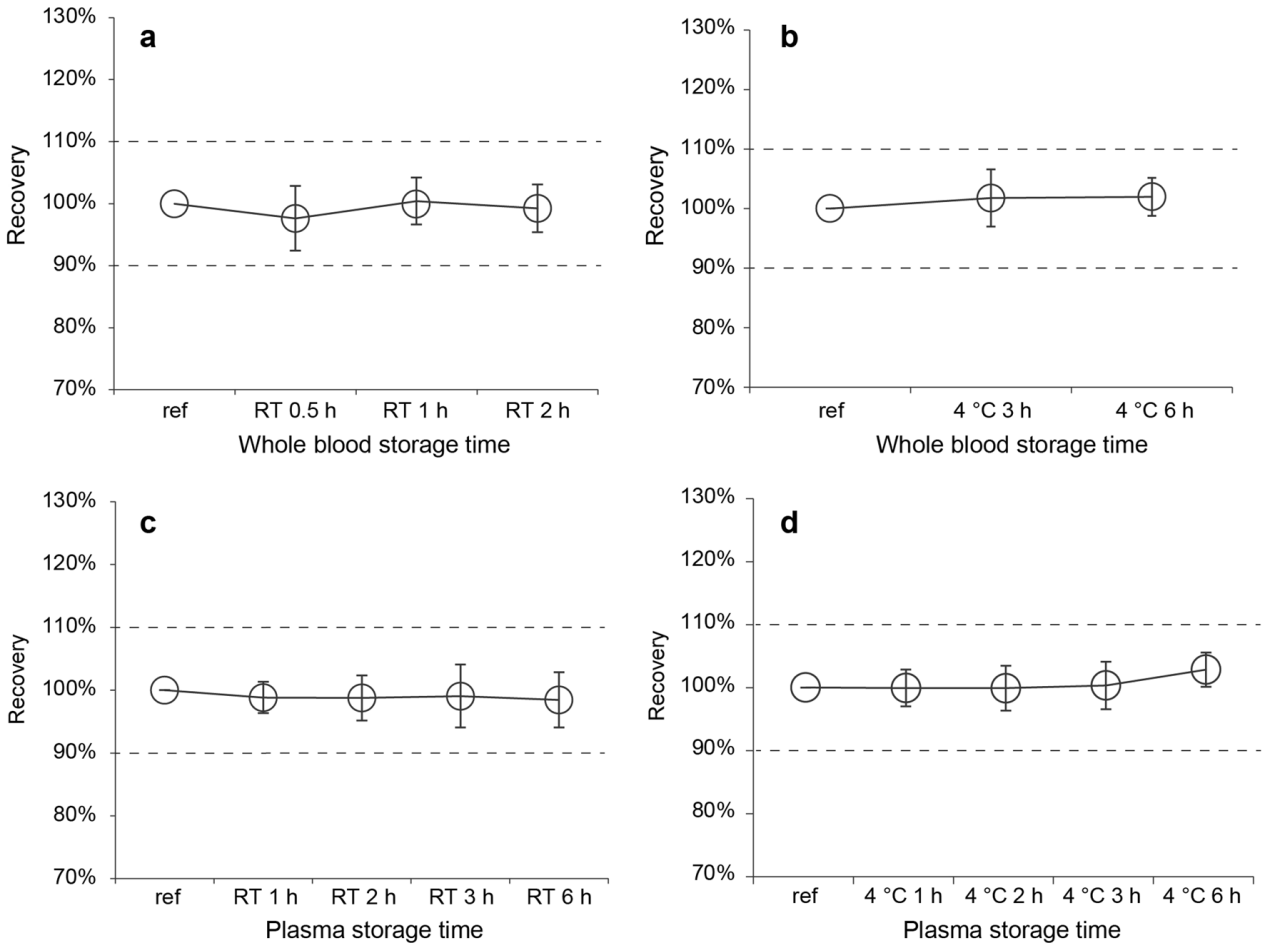
Recovery of the plasma Aβ42/Aβ40 level in different whole blood/plasma storage conditions. The effects of (**a**, **b**) whole blood and (**c**, **d**) plasma storage time at RT and 4 °C on the plasma Aβ42/Aβ40 level were evaluated. Plots and error bars indicate the mean values and standard deviations for 10 plasma samples. The y-axis shows recovery calculated as the percentage of the Aβ42/Aβ40 level obtained in each condition compared with that obtained in the reference condition. The black dashed lines show the borders of ± 10%. Aβ, β-amyloid; RT, room temperature; ref, reference.

The plasma Aβ42/Aβ40 levels were within the acceptable range when the WB samples were stored for 2 h at RT or 6 h at 4 °C (Fig. 2a,b). Plasma Aβ40 and Aβ42 levels exceeded a 10% difference from the reference value when the WB samples were stored for 2 h at RT (Supplementary Fig. S1). Following plasma separation, longer storage time of the plasma samples at RT tended to reduce Aβ40 and Aβ42 recovery (Supplementary Fig. S1). The mean differences in plasma Aβ40 and Aβ42 levels for 6 h at RT and 4 °C were 7.1 ± 4.0%, and 8.6 ± 4.5%, respectively. Since the trends of variation in Aβ40 and Aβ42 values were similar, the Aβ42/Aβ40 level met the criterion for acceptable recovery under these conditions (Fig. 2c,d). However, plasma Aβ42 levels decreased more than Aβ40 levels during storage at RT for 12 h, and the plasma Aβ42/Aβ40 level decreased slightly at RT (Supplementary Fig. S2).

### Effects of freezing and storage conditions

**Figure 3:**
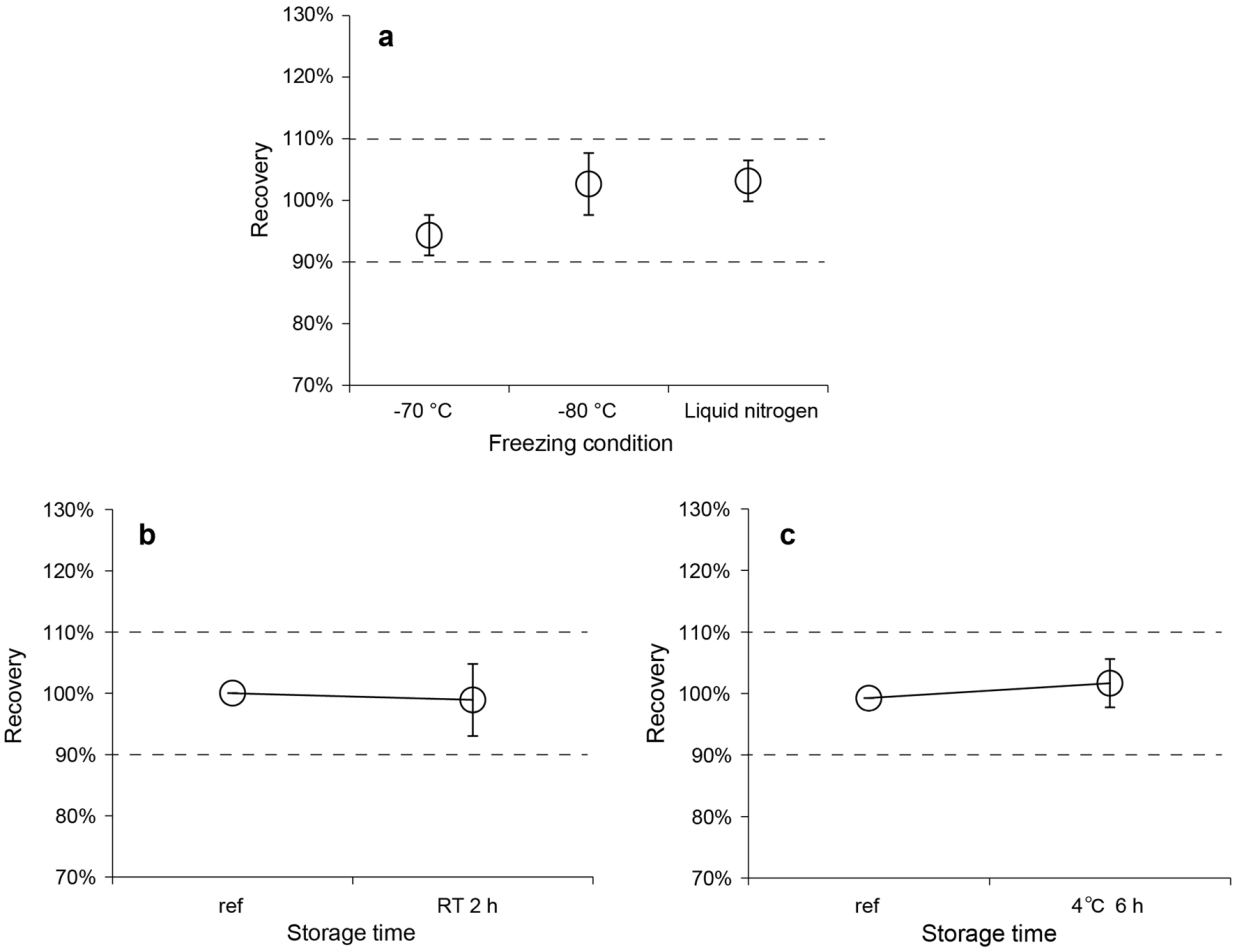
Recovery of the plasma Aβ42/Aβ40 level in different freezing/storage conditions. The effects of the (**a**) freezing condition and freezing timing of plasma samples stored at (**b**) RT or (**c**) 4 °C on the plasma Aβ42/Aβ40 level were evaluated. Plasma samples were frozen in deep freezers at − 70 °C or − 80 °C, or in liquid nitrogen. Plots and error bars indicate the mean values and standard deviations for 10 plasma samples. The y-axis shows recovery calculated as the percentage of the Aβ42/Aβ40 level obtained in each condition compared with that obtained in the reference condition. The black dashed lines show the borders of ± 10%. Aβ, β-amyloid; RT, room temperature; ref, reference.

There were no marked differences in the plasma Aβ42/Aβ40 level among freezing conditions (Fig. 3a). However, plasma Aβ40 and Aβ42 levels exceeded a 10% difference from the reference for all freezing conditions (Supplementary Fig. S3). Freezing after 2 h at RT and 6 h at 4 °C did not affect the recovery of Aβ40 and Aβ42 as well as the Aβ42/Aβ40 level (Fig. 3b,c, Supplementary Fig. S3).

The effects of individual pre-analytical parameters on plasma Aβ42/Aβ40 level are summarized in Table 1. We observed that plasma Aβ42/Aβ40 recovery was acceptable under all evaluated conditions.

**Table 1:** Summary of the effects of individual pre-analytical parameters on plasma Aβ42/Aβ40 levels.

Evaluated condition	Pre-analytical parameter	Acceptable condition (Aβ42/Aβ40 results)
WB stability	Time from WB collection to plasma separation, different temperatures	Up to 2 h at RT (Fig. 2a) Up to 6 h at 4 °C (Fig. 2b)
Plasma stability	Time from plasma separation to measurement, different temperatures	Up to 6 h at RT (Fig. 2c) Up to 6 h at 4 °C (Fig. 2d)
Freezing conditions	Different freezing temperatures/conditions for plasma samples	At − 70 °C, − 80 °C, or frozen using liquid nitrogen (Fig. 3a)
	Different freezing timings for plasma samples	Up to 2 h at RT (Fig. 3b) Up to 6 h at 4 °C (Fig. 3c)

*Aβ* β-amyloid, *WB* whole blood, *RT* room temperature.

### Combined effects of WB and plasma storage conditions

To verify plasma Aβ levels in clinical practice, sample-handling procedures should be determined considering the combined effects of the pre-analytical parameters. This is because potential interactions between these parameters may result in unacceptable plasma Aβ levels. Therefore, we investigated the combined effects of these factors on plasma Aβ42/Aβ40 levels. Additionally, in consideration of sample handling in clinical practice, we evaluated the following pre-analytical conditions: (1) WB centrifugation was performed at RT instead of 4 °C, (2) plasma samples were frozen at − 20 °C in addition to below − 70 °C, and (3) plasma Aβ levels were measured within 6 h at 4 °C instead of 0.5 h at RT after thawing plasma samples.

**Figure 4:**
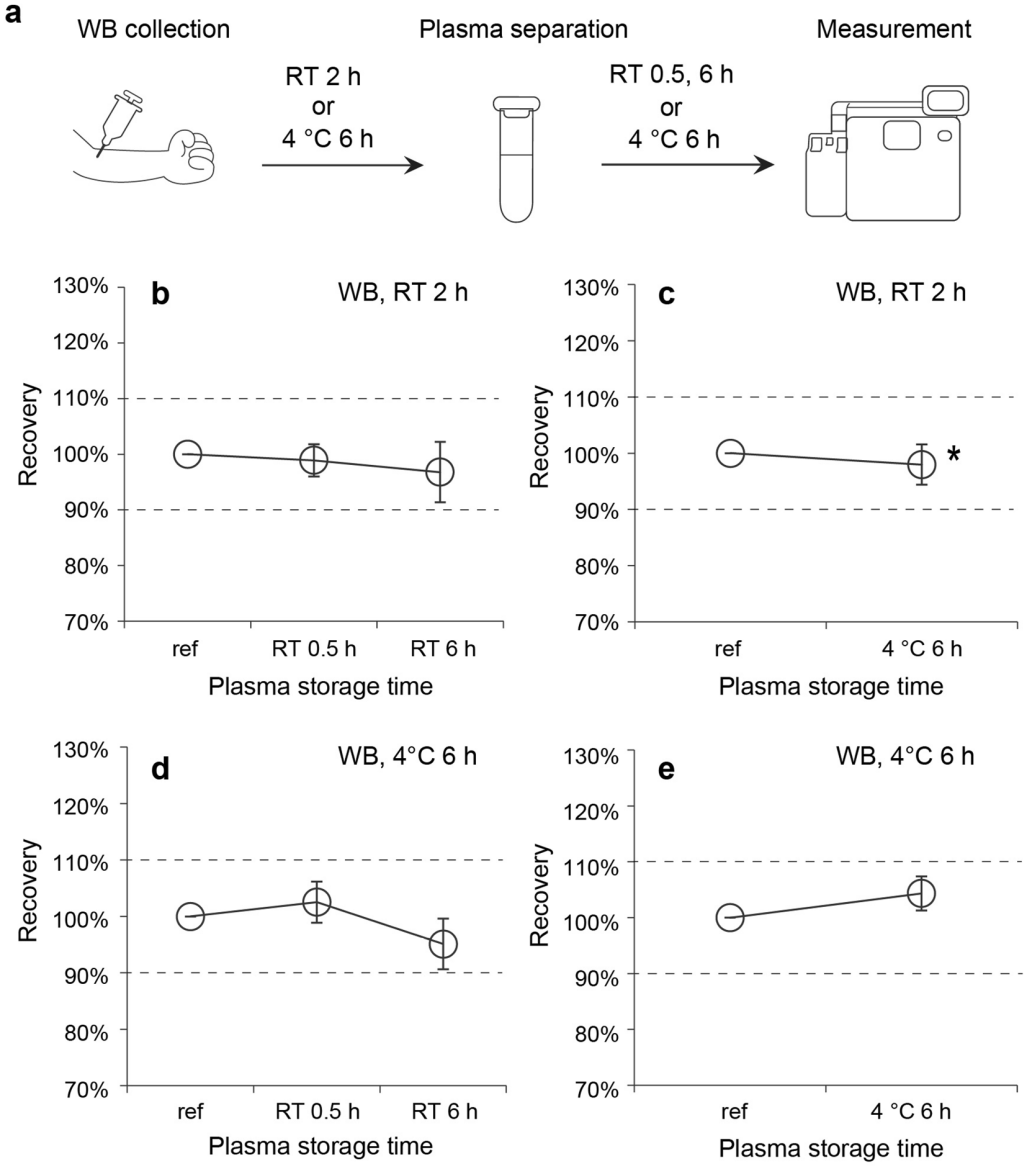
Combined effects of WB and plasma storage conditions on the plasma Aβ42/Aβ40 level. (**a**) Schematic of sample handling from WB collection to measurement. WB samples were stored for 2 h at RT or 6 h at 4 °C. Effects of plasma storage time and temperature on the Aβ42/Aβ40 level under WB storage conditions stored for (**b**, **c**) 2 h at RT or (**d**, **e**) 6 h at 4 °C. Plots and error bars indicate the mean values and standard deviations for 10 plasma samples. The condition indicated with an asterisk was analyzed using only five plasma samples because of insufficient plasma sample volumes. The y-axis shows recovery calculated as the percentage of the Aβ42/Aβ40 level obtained in each condition compared with that obtained in the reference condition. The black dashed lines show the borders of ± 10%. Aβ, β-amyloid; WB, whole blood; RT, room temperature; ref, reference.

We first evaluated the combined effects of WB and plasma storage conditions, and the experimental conditions for these combinations are shown in Fig. 4a. We selected the longest WB and plasma storage time from our evaluation of individual pre-analytical parameters for these experiments (Fig. 2). When WB was stored at RT for 2 h, plasma Aβ42/Aβ40 levels were acceptable, irrespective of whether plasma was stored at RT for 0.5–6 h or at 4 °C for 6 h (Fig. 4b,c). A similar result was obtained when the storage condition of WB was changed to 6 h at 4 °C (Fig. 4d,e). These evaluations were performed by WB centrifugation at RT, instead of 4 °C, which was previously assessed, suggesting that the centrifugation temperature does not affect plasma Aβ42/Aβ40 levels. Furthermore, plasma Aβ40 and Aβ42 levels did not change under either WB storage condition for up to 6 h of plasma storage at 4 °C, but they tended to decrease with RT storage (Supplementary Fig. S4).

### Combined effects of WB and plasma storage and freezing conditions

**Figure 5:**
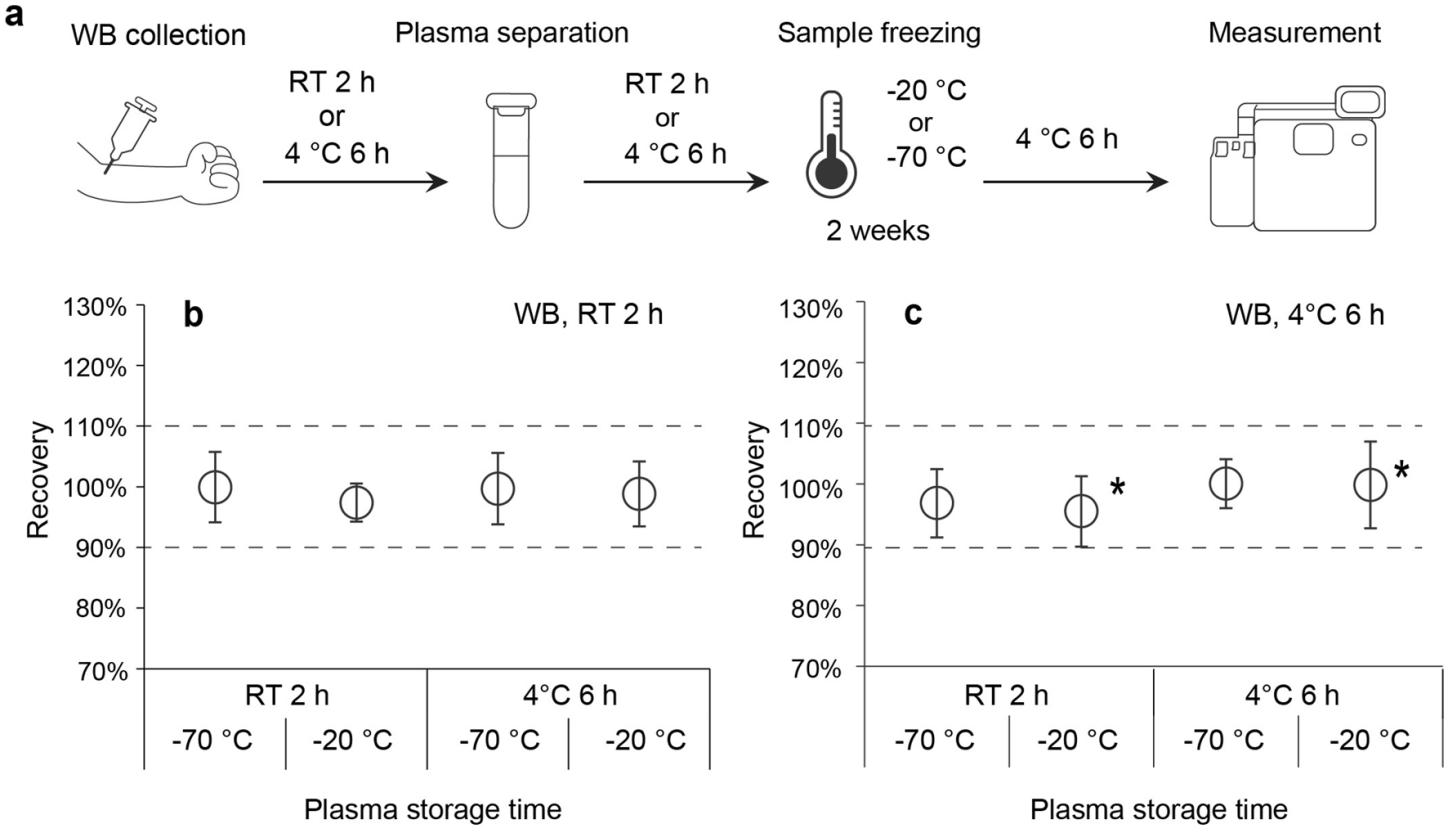
Combined effects of WB and plasma storage and freezing conditions on the plasma Aβ42/Aβ40 level. (**a**) Schematic of sample handling from WB collection to measurement. WB samples were stored for (**b**) 2 h at RT or (**c**) 6 h at 4 °C. Plasma samples were frozen at − 20 °C or − 70 °C for 2 weeks after plasma storage for 2 h at RT or 6 h at 4 °C. Effects of plasma storage time and temperature on the Aβ42/Aβ40 level under WB storage conditions b and c. Plots and error bars indicate the mean values and standard deviations for 10 plasma samples. The y-axis shows recovery calculated as the percentage of the Aβ42/Aβ40 level obtained in each condition compared with that obtained in the reference condition. In the conditions indicated with an asterisk in (**c**), four (4 °C 6 h, − 20 °C) or five (RT 2 h, − 20 °C) plasma samples were excluded from the analysis because of the generation of fibrin clots with supercooling. The black dashed lines show the borders of ± 10%. Aβ, β-amyloid; WB, whole blood; RT, room temperature.

It is assumed that in addition to measurements in non-frozen conditions, measurements in frozen plasma will often be performed in clinical practice. Therefore, we evaluated the combined effects of WB and plasma storage conditions under freezing conditions. The experimental conditions for these combinations are shown in Fig. 5a. In this evaluation, all frozen samples were thawed by 1-h incubation at RT, followed by 6 h of storage at 4 °C, instead of quick measurement after thawing, so that the evaluation would be conducted under harsher and more practical conditions.

Regardless of the combination of storage conditions for WB (RT for 2 h or 4 °C for 6 h), subsequent storage conditions for plasma (RT for 2 h or 4 °C for 6 h), or freezing conditions (− 20 °C or − 70 °C), Aβ42/Aβ40 levels were acceptable (Fig. 5b,c). However, plasma concentrations of Aβ40 and Aβ42 tended to decrease (Supplementary Fig. S5).

### An appropriate sample-handling procedure for measuring plasma Aβ levels using the HISCL analyzer

**Figure 6:**
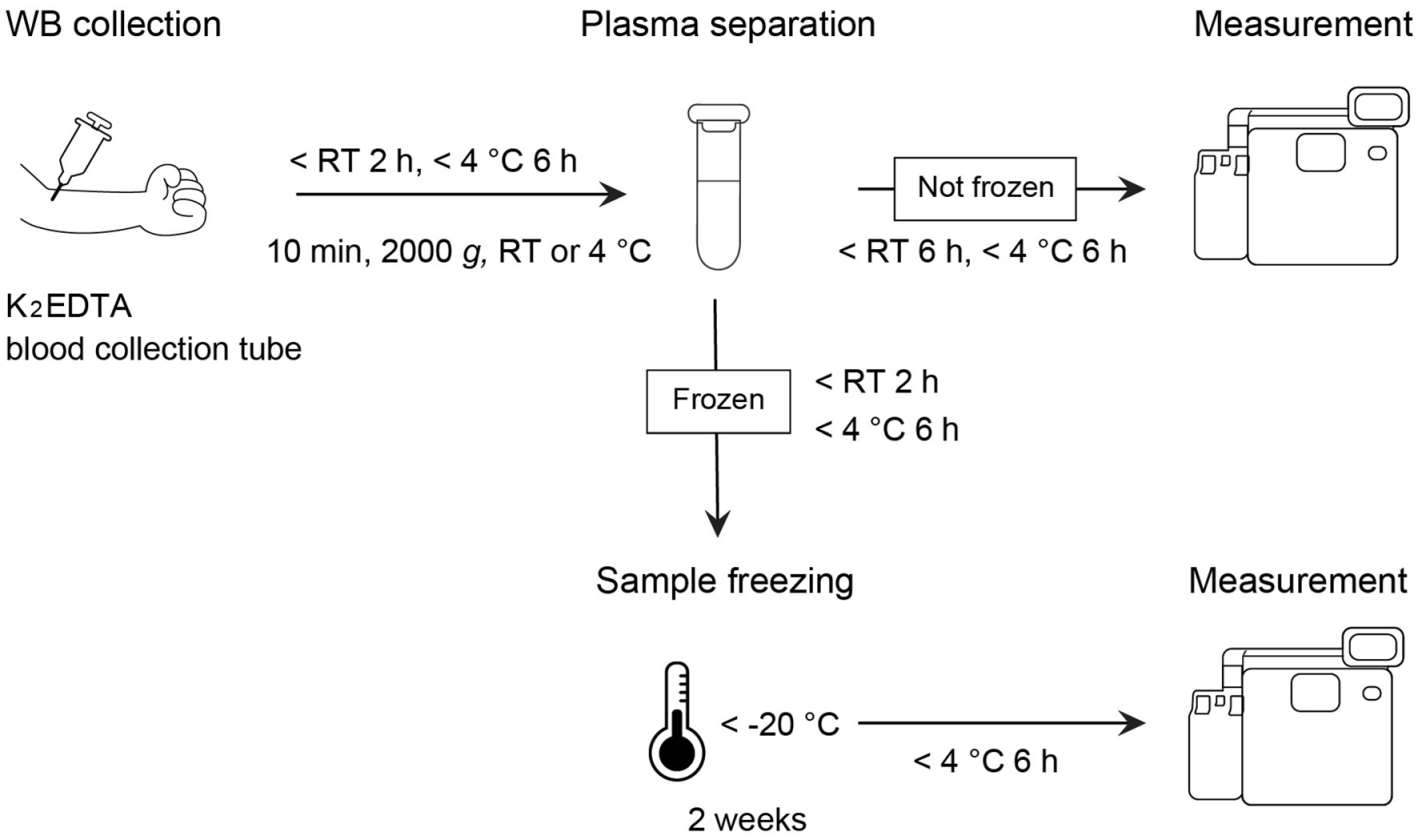
The proposed appropriate sample-handling procedure from WB collection to measurement of plasma Aβ42/Aβ40 levels. Liquid nitrogen may also be used to freeze plasma samples. Aβ, β-amyloid; EDTA, ethylenediaminetetraacetic acid; WB, whole blood; RT, room temperature.

On the basis of these results, we established a sample-handling procedure for measuring plasma Aβ42/Aβ40 levels using the HISCL analyzer (Fig. 6). This appropriate procedure includes additional conditions that met the criteria of the plasma Aβ42/Aβ40 level when evaluating the combined effects. Plots of Aβ42/Aβ40 values obtained under each condition are shown in Figures S6–S9.

## Discussion

In this study, we investigated the effects of pre-analytical parameters on the plasma Aβ42/Aβ40 level and proposed an appropriate sample-handling procedure for measuring plasma Aβ levels using the fully automated HISCL analyzer. Various analytical platforms have been used to evaluate the effects of pre-analytical parameters on plasma Aβ quantification. Verberk et al.^29^ compared the effects of pre-analytical factors on plasma biomarker levels such as Aβ40 and Aβ42 obtained from six different analytical platforms, including mass spectrometry (MS) methods (e.g., the C2N MS assay) and digital enzyme-linked immunosorbent assays (ELISA; e.g., the single-molecule array[SIMOA]). In this study, plasma-handling procedures were recommended for the six analytical platforms used to measure AD biomarkers, including Aβ42/Aβ40. However, the degree of pre-analytical effects (e.g., WB/plasma storage time) on plasma Aβ42/Aβ40 levels differed between Aβ assays. For example, the Aβ42/Aβ40 level measured using a matrix-assisted laser desorption/ionization time-of-flight MS assay was more significantly affected by WB/plasma storage conditions than that obtained using other assays (e.g., C2N MS, SIMOA, and EUROIMMUN ELISA assay)^29^. This result is not surprising since different analytical platforms vary in sample-handling factors such as stirring speed and reaction time/temperature. Consequently, the effects of pre-analytical parameters on plasma Aβ42/Aβ40 levels may differ between analytical platforms. Therefore, establishing standard recommendations for sample handling specifically using the HISCL analyzer is important.

We first clarified the independent effects of pre-analytical parameters such as sample storage time/temperature and freezing conditions on plasma Aβ levels measured by the HISCL analyzer. We observed that the plasma Aβ42/Aβ40 level was acceptable under all conditions. However, the plasma Aβ40 and Aβ42 levels exceeded the ± 10% allowed difference under some conditions, especially longer plasma storage time at RT. Our additional study suggested that plasma Aβ42 levels decreased more than Aβ40 levels for storage at RT for over 6 h and that the plasma Aβ42/Aβ40 level decreased slightly at RT. Previous studies extended the time of WB centrifugation delay up to 24 h and showed that the recovery rates according to the plasma Aβ42/Aβ40 level were lower at RT than at 4 °C^27,29,30^. These results highlight that 4 °C may be preferable to RT for sample storage. The Aβ molecule, particularly Aβ42, is unstable in plasma as it is hydrophobic. A possible explanation for the decrease in the Aβ42/Aβ40 level is the formation of self-aggregated Aβ42 peptides^33^. The hydrophobic property of the Aβ peptide itself may also trigger non-specific binding to hydrophobic surfaces as well as Aβ aggregation. Therefore, the decreased Aβ42/Aβ40 level could also be attributed to the adsorption of Aβ42 to the inner tube/tip surface by hydrophobic interaction. In any case, further evaluation of Aβ peptide stability in plasma, focusing on structural and molecular properties, is needed. Plasma Aβ-aggregate assays (oligomer and/or fibrils) will likely help in understanding the behavior of Aβ molecules in plasma.

In addition, our results highlight that the plasma Aβ42/Aβ40 level may remain stable despite the observed effects of pre-analytical factors on individual Aβ40 and Aβ42 levels. Freezing conditions affected plasma Aβ40 and Aβ42 recovery, resulting in unacceptable values; however, no marked differences in the plasma Aβ42/Aβ40 level were observed. Binette et al.^34^ reported the effects of confounding factors on AD plasma biomarkers. Although plasma Aβ40 and Aβ42 levels were affected by confounding factors such as creatinine and body mass index, these changes were canceled out by calculating the Aβ42/Aβ40 level. Plasma Aβs are derived from the brain as well as peripheral tissues and cleared by the kidney and liver. Therefore, plasma Aβ40 and Aβ42 levels may affect internal (e.g., Aβ production/clearance functions) as well as external (e.g., pre-analytical sample handling, type of analytical platform) factors. This is one reason why the plasma Aβ42/Aβ40 level, rather than Aβ40 and Aβ42 levels, has been widely investigated and shown to predict brain Aβ pathology characterized by CSF and/or PET status with high accuracy^21,24–26,35^. In real-world settings, the plasma Aβ42/Aβ40 level would be used for assisting AD diagnosis with CSF and PET testing. Therefore, we aimed to establish acceptable plasma Aβ42/Aβ40 levels by controlling the sample-handling procedure.

A strength of our study is that we determined our sample-handling procedure by investigating the combined effects of pre-analytical conditions on the plasma Aβ42/Aβ40 level. Many studies have provided recommendations by evaluating individual pre-analytical parameters without considering the interactions between them. We showed that the plasma Aβ42/Aβ40 level was within acceptable ranges even when pre-analytical variables were changed in combination. These results suggest no interaction between pre-analytical parameters that affects Aβ42/Aβ40 levels under our assessed conditions. Our recommended protocol is similar to others that have not evaluated interactions between each pre-analytical parameter and plasma Aβ42/Aβ40 levels^27,29,30^. However, since we developed our protocol to account for real-world application, our plasma Aβ assay combined with the appropriate use of this sample-handling protocol may be feasible for implementation in routine clinical practice.

The current study had some limitations. The freezing period of 2 weeks is short in terms of research purposes, particularly with regard to the use of retrospective samples such as historical biobank cohorts. The sample handling protocol tested in this study is intended for clinical practice use. Therefore, only the evaluation of samples frozen for a relatively short period of time has been performed, and the long-term storage stability of the samples has not been evaluated. In order to confirm the long-term stability, it is necessary to evaluate such stability analysis. Another study limitation is that our protocol was established and validated by evaluating plasma samples obtained from healthy volunteers. There is a difference in Aβ42/40 values between the healthy individuals assessed in the current study and previously reported AD disease groups^26^. Therefore, the % change in plasma Aβ42/40 levels may differ in subjects with AD pathology present in the brain. Hence, additional studies are needed to determine the applicability of this protocol to diverse patients with different pathologies.

In conclusion, by evaluating the effects of individual and combined pre-analytical parameters on plasma Aβ levels, we established a recommended sample-handling procedure for obtaining Aβ42/Aβ40 levels calculated from HISCL analyzer measurements. We believe that our plasma Aβ assay coupled with our sample-handling recommendation is a promising candidate for aiding AD diagnosis in routine clinical practice.

## Methods

### Blood sampling

Blood samples were obtained from healthy volunteers at Seishin Kenshin Center (Kobe, Japan). This study was approved by the Sysmex Ethics Committee (approval number 2019–047) and conducted in accordance with the tenets of the Declaration of Helsinki. All participants provided informed consent for their blood samples to be used in this study. Ten blood samples from healthy volunteers were used to evaluate the effects of one pre-analytical condition on plasma Aβ levels. Whole blood (WB) samples were collected in 6 mL dipotassium ethylenediaminetetraacetic acid tubes (BD Vacutainer® Blood Collection Tubes, cat. No. 365900; Becton Dickinson, Franklin Lakes, NJ, USA, and stored at 4 °C until centrifugation (2000 g, 10 min, 4 °C), which was performed within 1 h of collection. After plasma separation, the samples were stored in polypropylene tubes (Bio-Bik, cat. No. 2200; Ina-Optica, Osaka, Japan) at room temperature (RT), and plasma Aβ40 and Aβ42 levels were measured within 30 min at RT; this was set as the reference condition.

### Plasma measurement

Plasma Aβ40 and Aβ42 levels were quantified using the HISCL analyzer. Each measurement required 30 μL of plasma, and measurements were completed within 17 min per assay. In this study, Aβ assays that previously showed high analytical and clinical performance were used^26,36^.

### Evaluation of pre-analytical parameters

We compared the effects of individual pre-analytical parameters on the plasma Aβ42/Aβ40 level with that determined under the reference condition. The effects of individual parameters were evaluated by changing one variable from the reference condition at a time. The following parameters were assessed: (1) storage time of WB samples at different temperatures, (2) storage time of plasma samples from plasma separation to measurement, (3) freezing timing after plasma separation, and (4) freezing temperature (Fig. 1). The plasma samples were transferred to freezers set at − 70 °C or − 80 °C, or frozen using liquid nitrogen within 30 min of plasma separation at RT. Freezing timing means the time from plasma separation to freezing samples using liquid nitrogen. The frozen plasma samples were thawed for 1 h at RT. After evaluation of the effects of individual parameters on the plasma Aβ42/Aβ40 level, the combined effects of these parameters were determined. In the assessment of the combined effects of WB/plasma stability, we selected the longest time from each individual pre-analytical test. Additionally, the following conditions were added to the evaluation of combined effects: (1) centrifugation at 2000 g for 10 min at RT, (2) freezing at − 20 °C, and (3) storage time for 6 h after thawing the plasma samples. For frozen samples, measurements were performed after 2 weeks’ storage of plasma samples in the freezers set at − 20 °C or − 70 °C.

### Data analysis

Measurement of Aβ40 and Aβ42 levels was performed in triplicate for each individual, and the average per individual was obtained. To evaluate the effect of each pre-analytical parameter, plasma Aβ40 and Aβ42 levels from 10 individuals were measured, and the mean Aβ42/Aβ40 level and standard deviation (SD) was determined. Acceptable plasma Aβ42/Aβ40 levels were defined as those that differed by less than ± 10% from that obtained in the reference condition, as described previously^27^. Therefore, we determined whether the mean ± SD for each test was within ± 10% of the reference plasma Aβ42/Aβ40 level.

### Supplementary Information

Supplementary Figures.

## Data Availability

The data and materials obtained and/or analyzed during the study are not publicly available because of ethical concerns but are available from the corresponding author upon reasonable request.

## Abbreviations

Aβ: β-Amyloid
AD: Alzheimer’s disease
CSF: Cerebrospinal fluid
ELISA: Enzyme-linked immunosorbent assay
MS: Mass spectrometry
PET: Positron emission tomography
RT: Room temperature
SD: Standard deviation
WB: Whole blood
