# Supplementary material for: The appropriate sample-handling procedure for measuring the plasma β-amyloid level using a fully automated immunoassay
Source: Sci Rep. 2024 Jun 20;14:14266. doi: 10.1038/s41598-024-65264-1 (PMC11190145; doi:10.1038/s41598-024-65264-1)
Supplement: Supplementary file 1 — Supplementary Figures. [file 41598_2024_65264_MOESM1_ESM.docx]

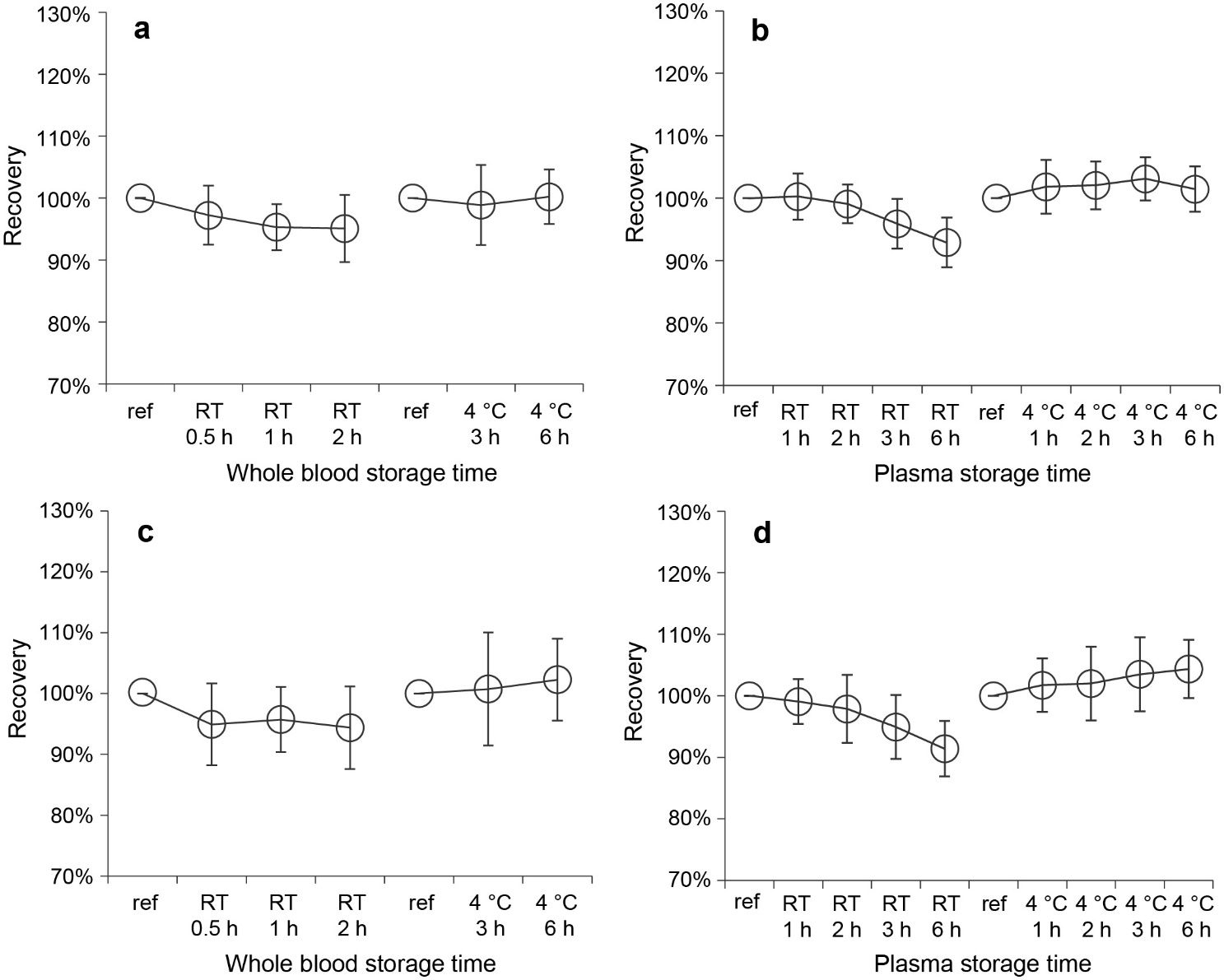


Figure S1. Recovery of plasma Aβ40 and Aβ42 levels in different whole blood/plasma storage conditions. The effects of storage time and temperature of whole blood and plasma on the plasma (a, b) Aβ40 and (c, d) Aβ42 levels were evaluated. Plots and error bars indicate the mean values and standard deviations for 10 plasma samples. The y-axis shows recovery calculated as the percentage of the Aβ40 or Aβ42 level obtained in each condition compared with those obtained in the reference condition. Aβ, β-amyloid; RT, room temperature; ref, reference


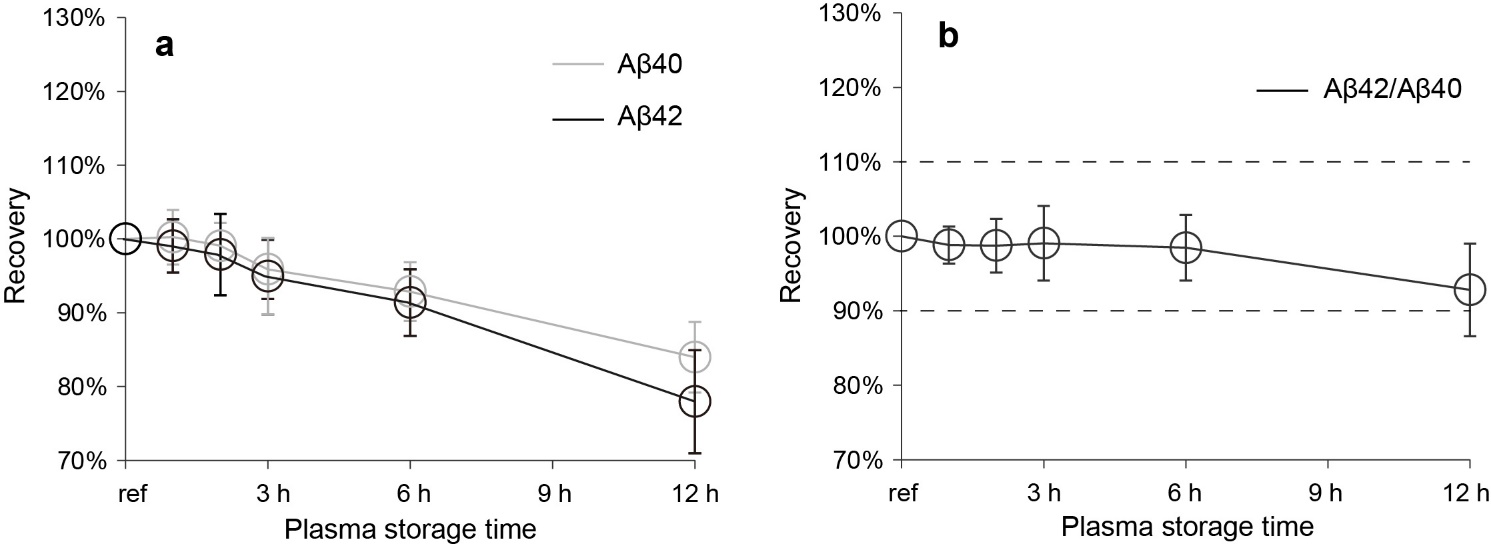


Figure S2. Effects of storage time and temperature on the plasma (a) Aβ40, Aβ42, and (b) Aβ42/Aβ40 levels. Plots and error bars indicate the mean values and standard deviations for 10 plasma samples. The y-axis shows recovery calculated as the percentage of Aβ40, Aβ42, or Aβ42/Aβ40 levels obtained in each condition compared with those obtained in the reference condition. Aβ, β-amyloid; ref, reference


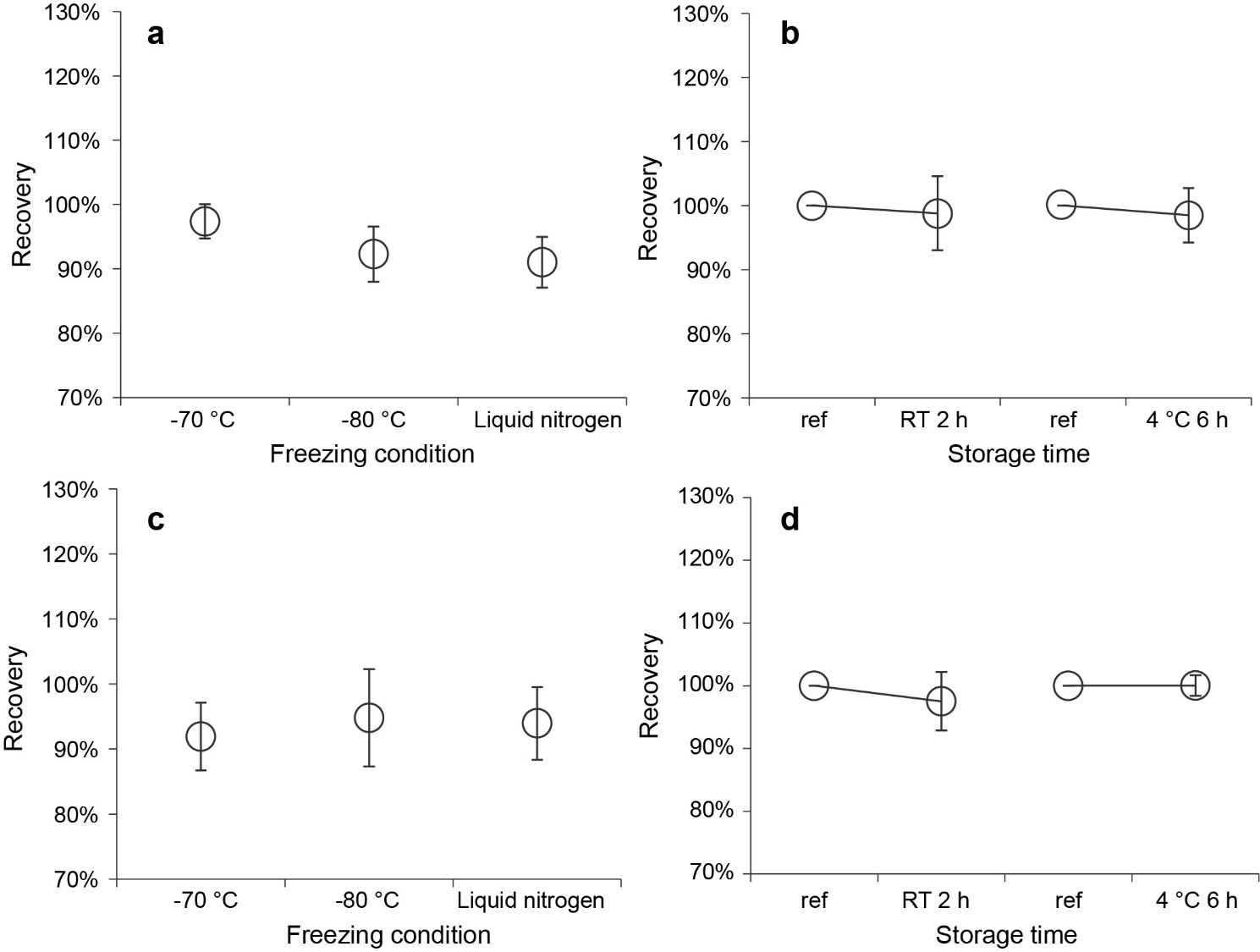


Figure S3. Recovery of plasma Aβ40 and Aβ42 levels in different freezing/storage conditions. The effects of freezing condition and storage temperature on the plasma (a, b) Aβ40 and (c, d) Aβ42 levels were evaluated. Plots and error bars indicate the mean values and standard deviations for 10 plasma samples. The y-axis shows recovery calculated as the percentage of the Aβ40 or Aβ42 level obtained in each condition compared with those obtained in the reference condition. Aβ, β-amyloid; RT, room temperature; ref, reference


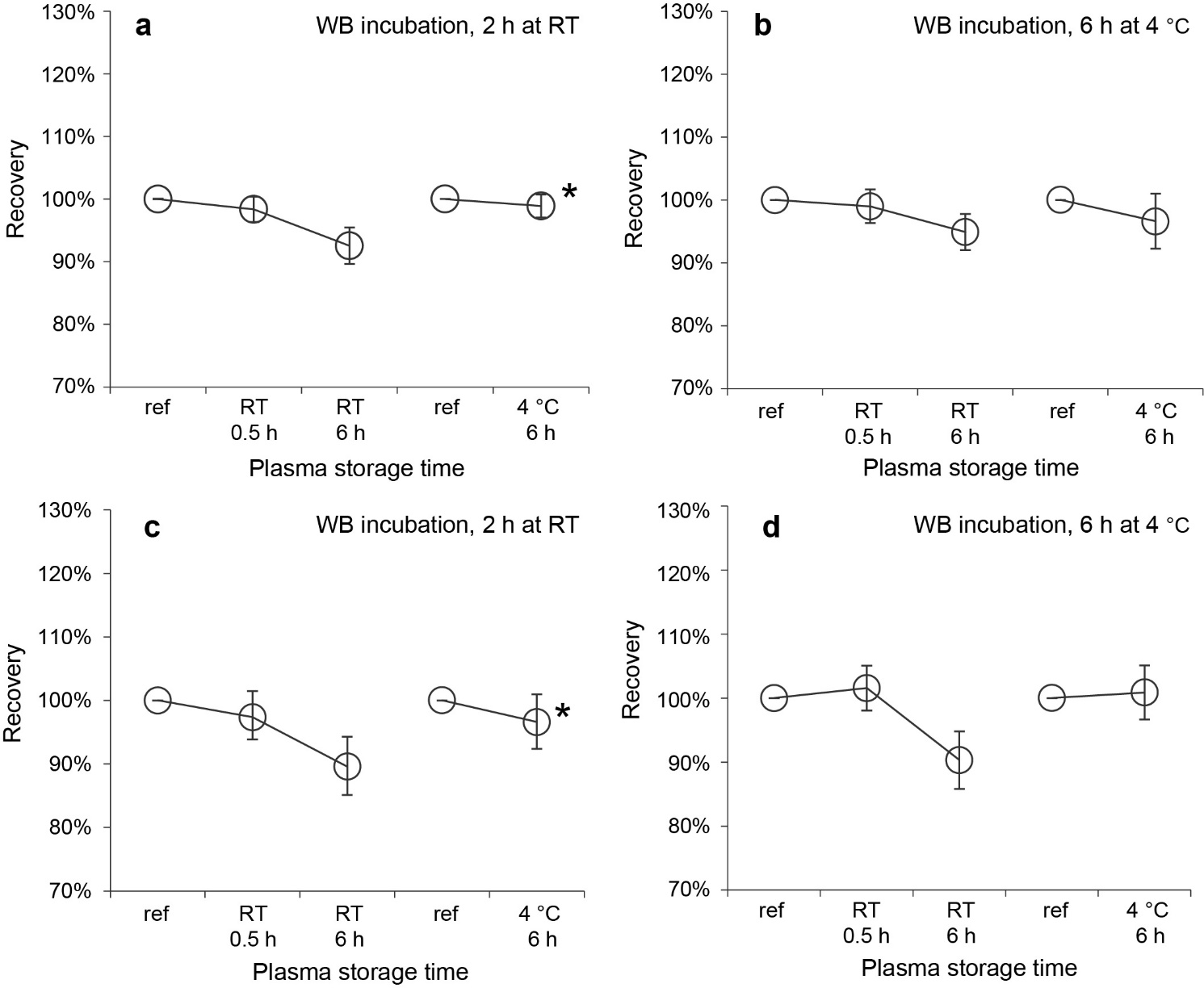


Figure S4. Recovery of plasma Aβ40 and Aβ42 levels in different WB and plasma storage conditions. The combined effects of WB and plasma storage time and temperature on the (a, b) Aβ40 and (c, d) Aβ42 levels were evaluated. WB samples were stored for 2 h at RT or 6 h at 4 ℃. Plots and error bars indicate the mean values and standard deviations for 10 plasma samples. The conditions indicated with asterisks were analyzed using only five plasma samples because of insufficient plasma sample volumes. The y-axis shows recovery calculated as the percentage of the Aβ40 or Aβ42 level obtained in each condition compared with those obtained in the reference condition. Aβ, β-amyloid; WB, whole blood; RT, room temperature; ref, reference


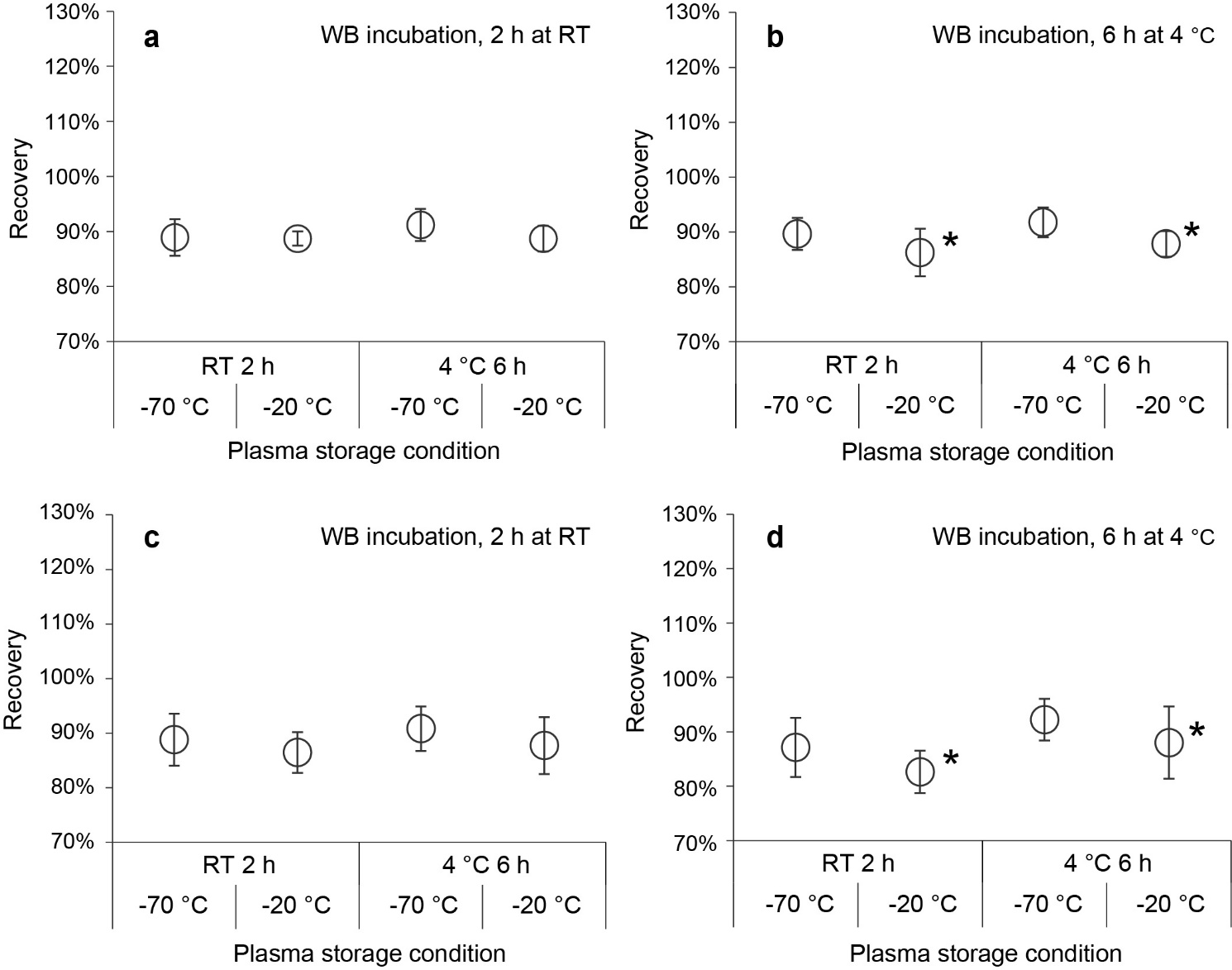


Figure S5. Combined effects of plasma storage time and temperature on the Aβ40 (a, b) and Aβ42 (c, d) levels. WB samples were stored for 2 h at RT or 6 h t 4 ℃. Plots and error bars indicate the mean values and standard deviations for 10 plasma samples. The y-axis shows recovery calculated as the percentage of the Aβ42 or Aβ40 level obtained in each condition compared with those obtained in the reference condition. In the conditions indicated with an asterisk, four (4 ℃ 6 h, −20 ℃) or five (RT 2 h, −20 ℃) plasma samples were excluded from the analysis due to the generation of fibrin clots with supercooling. Aβ, β-amyloid; WB, whole blood; RT, room temperature


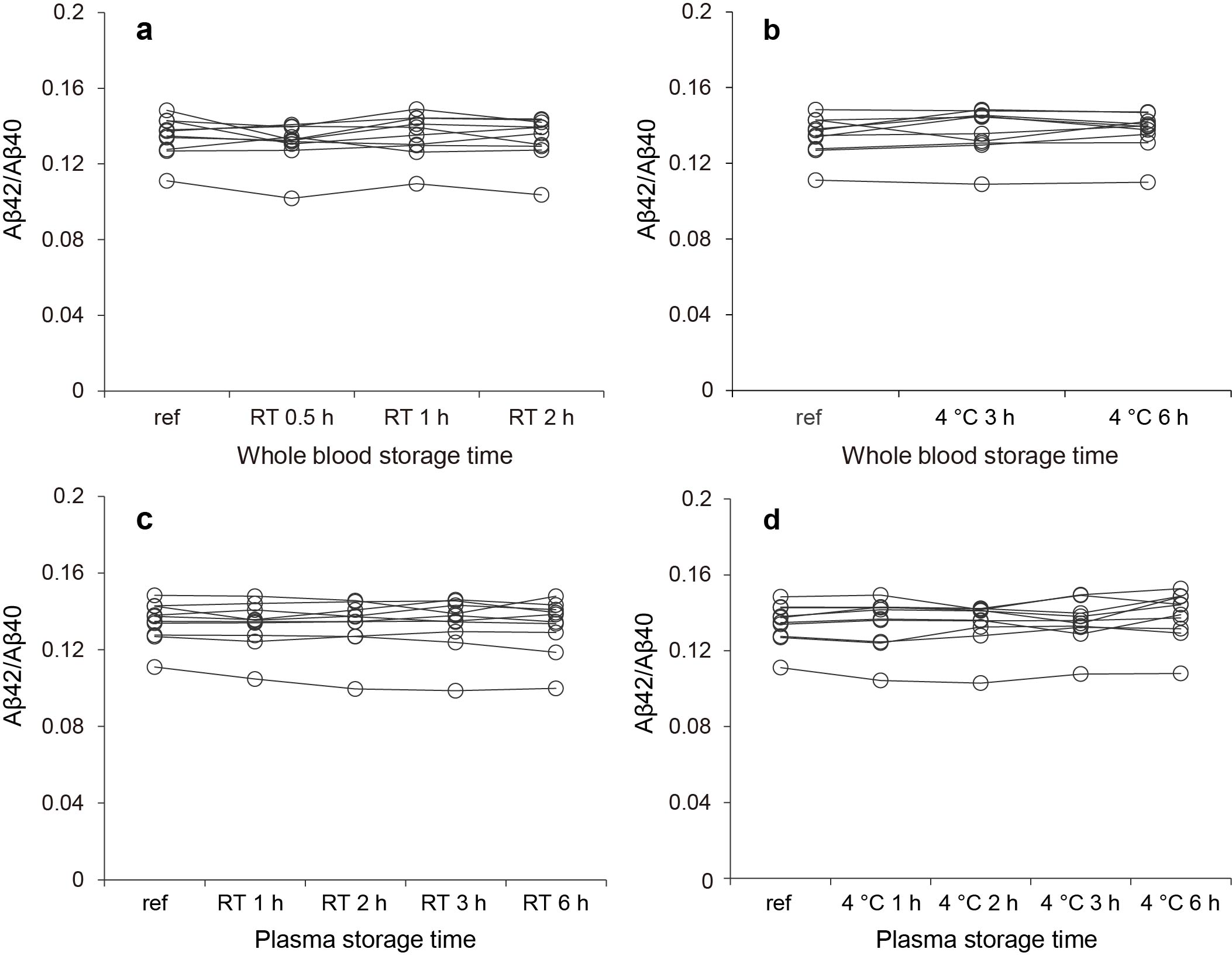


**Figure S6.** Plasma Aβ42/Aβ40 levels in whole blood/plasma samples stored under different conditions. The effects of (a, b) whole blood and (c, d) plasma storage time at RT and 4°C were evaluated. Plasma Aβ42/Aβ40 levels in ten individual samples are plotted in each figure.

Aβ, β-amyloid; ref, reference; RT, room temperature


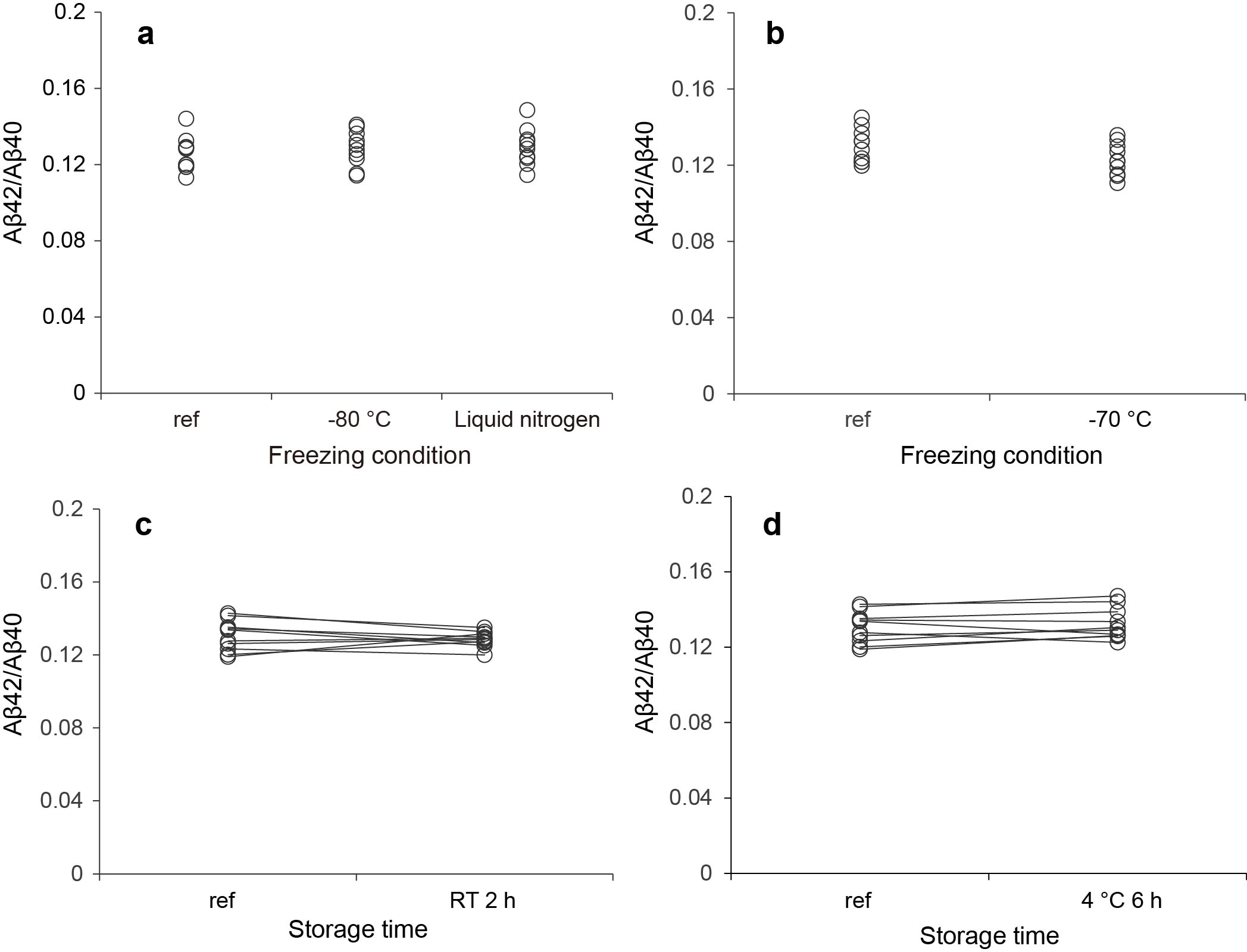


**Figure S7.** Plasma Aβ42/Aβ40 levels detected in samples subjected to different freezing/storage conditions. Effects of (a, b) freezing condition and freezing timing of plasma samples stored at (c) RT or (d) 4°C. Plasma samples were frozen at -70°C or -80°C, or in liquid nitrogen. Plasma Aβ42/Aβ40 levels in ten individual samples are plotted in each figure.

Aβ, β-amyloid; ref, reference; RT, room temperature


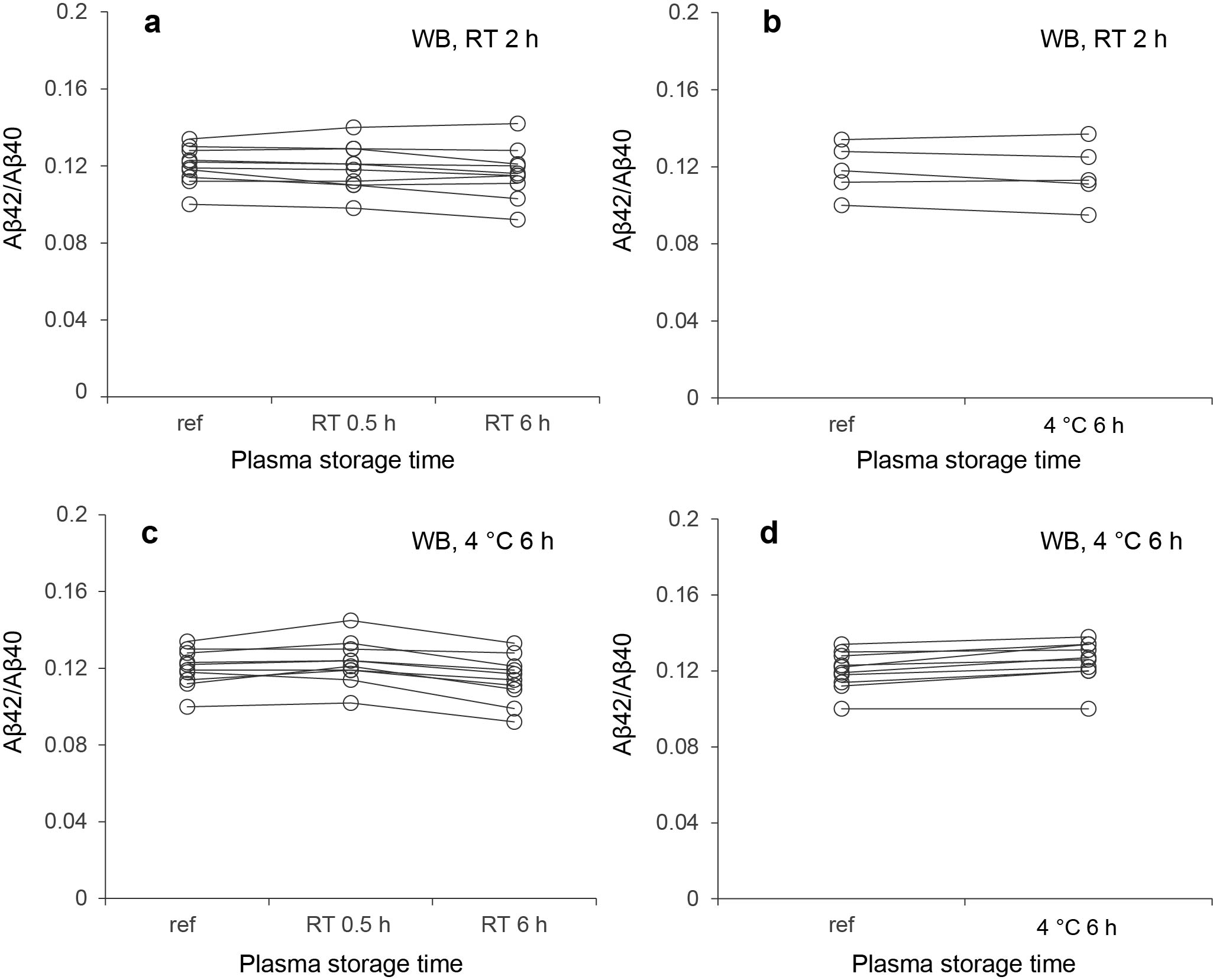


Figure S8. Combined effects of WB and plasma storage conditions on plasma Aβ42/Aβ40 levels. WB samples were stored for 2 h at RT or 6 h at 4°C. Effects of plasma storage time and temperature on Aβ42/Aβ40 levels in WB samples stored for (a, b) 2 h at RT or (c, d) 6 h at 4°C. Plasma Aβ42/Aβ40 levels in ten individual samples are plotted in each figure, with the exception of Figure S8b in which only five are plotted due to insufficient plasma sample volumes.

Aβ, β-amyloid; ref, reference; RT, room temperature


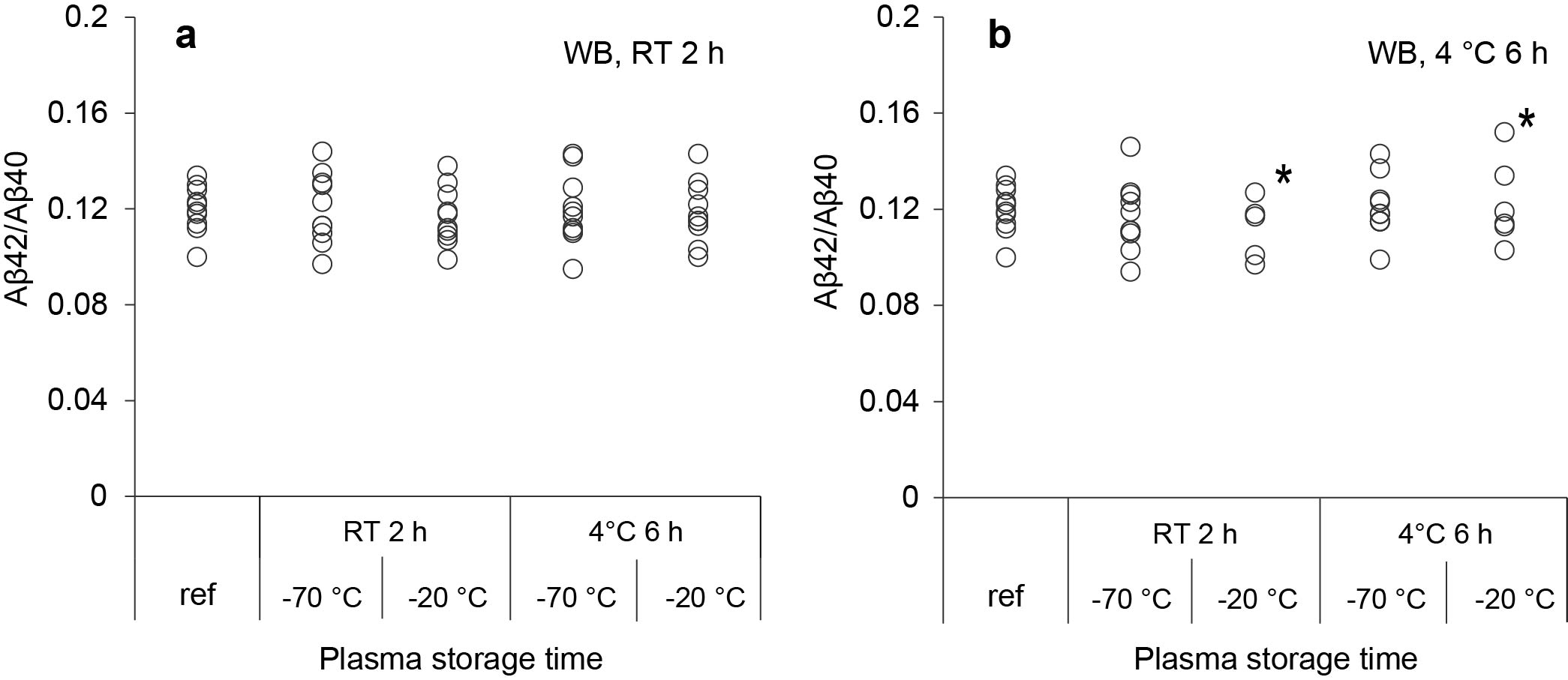


Figure S9. Combined effects of WB and plasma storage and freezing conditions on plasma Aβ42/Aβ40 levels. WB samples were stored for (a) 2 h at RT or (b) 6 h at 4°C. Plasma samples were frozen at -20°C or -70°C for 2 weeks after plasma storage for 2 h at RT or 6 h at 4°C. Plasma Aβ42/Aβ40 levels in ten individual samples are plotted in each figure, with the exception of Figure S9b, in which four (4°C 6 h, -20°C) or five (RT 2 h, -20°C) plasma samples were excluded from analysis due to the generation of fibrin clots with supercooling.

Aβ, β-amyloid; RT, room temperature; WB, whole blood
